# Vectored Immunoprophylaxis for Mucosal Immunity: Advances and Challenges Associated with Recombinant Secretory IgA Expression

**DOI:** 10.3390/vaccines14080692

**Published:** 2026-08-12

**Authors:** Benjamin J. Manchester, Jennifer L. Gommerman, Shayan Sharif, Leonardo Susta, Sarah K. Wootton

**Affiliations:** 1 Department of Pathobiology, University of Guelph, Guelph, ON N1G 2W1, Canada; bmanches@uoguelph.ca (B.J.M.);; 2 Department of Immunology, Temerty Faculty of Medicine, University of Toronto, 1 King’s College Circle, Toronto, ON M5S 1A8, Canada

**Keywords:** adeno-associated virus (AAV), vectored immunoprophylaxis, secretory IgA, mucosal immunity, monoclonal antibody, antibody engineering

## Abstract

Existing vectored immunoprophylaxis (VIP) approaches have primarily focused on IgG, which provides systemic protection but is less specialized in mucosal immunity. In contrast, secretory IgA (sIgA) plays a central role at epithelial surfaces, promoting pathogen neutralization while limiting inflammation. Although monoclonal IgA therapies are effective, their short half-life requires repeated dosing. Thus, VIP strategies enabling sustained sIgA expression at mucosal sites could transform mucosal infection prevention and treatment. This review outlines the key challenges associated with in vivo IgA expression and discusses critical considerations for VIP-mediated IgA delivery at mucosal surfaces, with emphasis on its potential for clinical translation. We provide a detailed overview of platforms for targeted IgA expression, including adeno-associated virus (AAV), adenoviral and lentiviral vectors, and lipid nanoparticle-based systems, alongside relevant routes of administration. Additionally, we examine emerging strategies to enhance the robustness, durability, and localization of IgA expression in vivo. Overall, VIP-enabled IgA expression represents an emerging strategy for enhancing mucosal immunity, with continued advances required to establish its role in the prevention and treatment of mucosal infections.

## 1. Introduction

The discovery of antibodies at the end of the 19th century marked a turning point in the understanding of how the body protects itself from infection [1]. In 1890, Emil von Behring and Shibasaburo Kitasato demonstrated that immunity to infection could be transferred between animals in the form of cell-free serum revealing the existence of protective factors in the serum later termed antibodies by Paul Ehrlich [1]. Although the existence of antibodies was known at the beginning of the 20th century, it was not until the improvement of protein electrophoresis techniques in the 1960s that the structure of these antibodies was elucidated and isotypes assigned [2]. It was during this time that a new class of immunoglobulin named IgA was identified as the prominent antibody in secretions such as saliva [3]. Subsequent studies showed that IgA is produced in abundance at mucosal surfaces, where it plays a major role in humoral mucosal immunity. In contrast, IgG is the predominant antibody in serum and tissue fluids and mediates systemic immune protection, although it can also contribute to immunity at select mucosal sites [3]. Further advancements in immunology in the 1970s led to the development of monoclonal antibodies in mice using a hybridoma method characterized by Kohler and Milstein in which therapeutic antibodies could be produced in mice [4]. However, initial clinical studies in humans were plagued by strong immune responses to foreign murine antibodies, limiting effectiveness of monoclonal antibody (mAb) treatments [4]. This led to the development of human–mouse chimeric antibodies and eventually humanized antibodies in the 1980s, making mAb treatments viable for the first time [5]. Monoclonal IgA treatments are a recent advancement and remain largely experimental, as challenges in large scale production have limited their clinical development [6]. Nonetheless, experimental studies have demonstrated their potential as therapeutics most notably the development of an IgA mAb specific for H5 hemagglutinin, which provided complete protection of mice challenged with the highly pathogenic avian H5N1 influenza virus [6]. Similarly, monoclonal IgA antibody treatments have shown strong protective effects in murine models of infection caused by *Campylobacter jejuni*, Enterotoxigenic *E. coli*, and *Salmonella enterica* serovar *Typhimurium* (*S. typhimurium*), further highlighting their promise as future therapeutics [7]. Despite the effectiveness of mAb treatments, a major limitation of these treatments is the need for repeat dosing due to the short half-life of IgA antibodies in serum [8], thereby highlighting the need for innovative approaches to achieve sustained IgA expression and targeted delivery at mucosal surfaces.

## 2. Biological Rationale for Using sIgA as a Therapeutic

### 2.1. Structure of IgA and Transcytosis at Mucosal Surfaces

**Figure 1:**
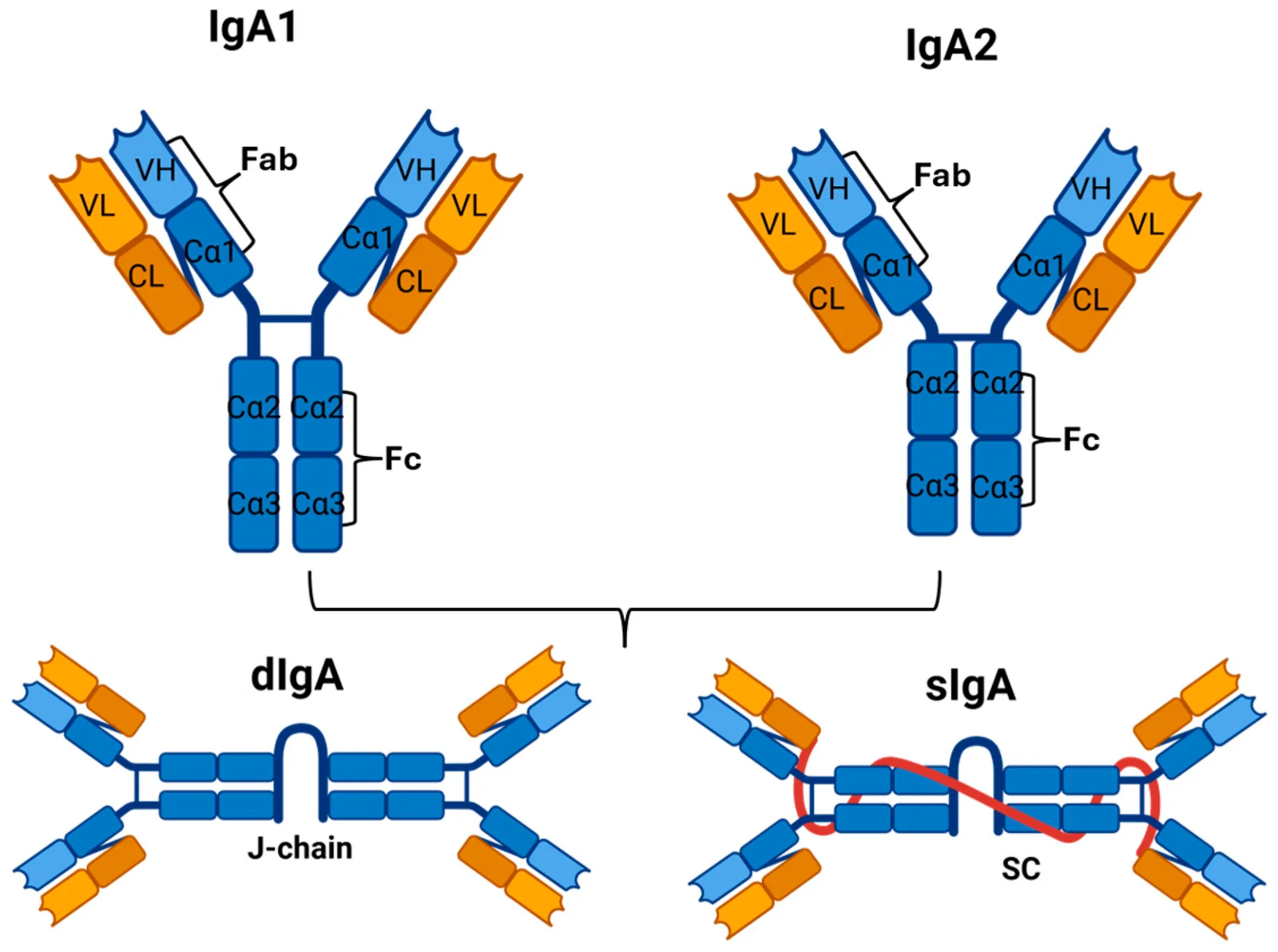
Schematic representation of the monomeric forms of the IgA1 and IgA2 antibody subclasses displaying the variable heavy (VH) and variable light (VL) chains, the constant light (CL) and constant heavy (Cα) chains, and the fragment antigen-binding (Fab) and fragment crystallizable sites (Fc). The polymeric forms of IgA are shown, including dimeric IgA (dIgA) in which two IgA monomers are linked by the joining chain (J-chain) and secretory IgA (sIgA), formed following the association of the dIgA with the secretory component (SC). Created in BioRender. Wootton, S. (2026) https://BioRender.com/v326eb1 (accessed on 1 July 2026).

IgA is the most highly produced antibody in the human body containing unique structural features that allow it to remain stable in harsh mucosal environments and provide immune protection at mucosal surfaces [9]. It has been calculated that a 70 kg human produces an estimated 4 g of IgA every day to cover the large surface area of mucosal epithelium present in the body [9]. Following the general structure of antibodies, IgA contains two identical light chains and two identical heavy chains containing variable Fragment antigen-binding (Fab) regions at the N-terminus that are antigen binding and constant Fragment crystallizable (Fc) regions that interact with immune receptors (see Figure 1) [10].

Each light chain consists of two globular domains, including a variable domain and constant domain in the Fab region of IgA [10]. The heavy chains consist of four globular domains, including a variable domain and constant domain Cα1 located in the Fab region followed by two constant domains Cα2 and Cα3 located in the Fc region of IgA (see Figure 1) [10]. IgA structure differs from other immunoglobulins in that it contains a hinge region between Cα1 and Cα2 that connects the Fab and Fc regions, providing flexibility to the antigen-binding arms, enabling more effective engagement with spatially diverse epitopes, promoting antigen crosslinking, and enhancing immune exclusion at mucosal surfaces [10]. Each globular domain of IgA forms a recognizable immunoglobulin-like fold characteristic of all antibodies consisting of 110 amino acids arranged in two beta-sheets composed of anti-parallel β-strands [10]. These beta-sheets are further stabilized by internal disulfide bonds between the variable domain of the heavy chain and variable domain of the light chain, Cα1, and the constant region of the light domain, along with the Cα2-Cα3 domains of both heavy chains [10]. There are two types of IgA antibodies found in humans including IgA1 and IgA2, with IgA1 being found primarily in serum and IgA2 being found in mucosal secretions such as intestinal mucus (see Figure 1) [11]. The IgA1 contains an extensive hinge region absent in IgA2, containing repeats of proline, threonine and serine with these residues containing multiple O-linked oligosaccharides [11]. This O-linked glycosylation on the hinge region is thought to modulate its conformation and increase flexibility but has also been found to make the region more susceptible to proteolysis [11]. Both subtypes of IgA contain N-linked oligosaccharides in the Cα2 domain, providing further structural integrity and protection from proteolysis [11].

The IgA secreted at mucosal surfaces primarily exists in a dimeric form in which two IgA monomers are covalently attached through a joining chain or J-chain polypeptide [12]. The J-chain is a small polypeptide of approximately 15 kDa and is produced by antibody-producing plasma cells in tandem with monomeric IgA in the lamina propria of mucosal surfaces [10]. The J-chain creates dimeric IgA (dIgA) using two specific cysteine residues that link the J-chain to the heavy chain of two IgA monomers [10]. This process is enhanced by the marginal zone B-1 cell-specific protein (MZB1) chaperone through the binding of the heavy chain of IgA, where the J-chain is bound preventing the degradation of dimeric IgA [13]. MZB1-deficient mice showed a measurable decrease in IgA production in response to external inflammatory stimuli highlighting the crucial role of MZB1 in promoting dIgA assembly [13]. The proper formation of dIgA is crucial for its interaction with the polymeric immunoglobulin receptor (pIgR) present on the basolateral surface of mucosal epithelial cells [14]. The pIgR receptor is a type I transmembrane protein consisting of an extracellular region that protrudes from the basolateral surface of epithelial cells, a single membrane spanning region and a cytoplasmic tail [14]. The extracellular ligand region of the pIgR comprises five domains, with domain 1 having three separate complementary determining regions (CDRs) that function to form covalent bonds with dIgA produced from plasma cells [14]. Domains 2 to 4 of the pIgR increase the binding efficiency of the receptor by creating the optimal spacing between domains 1 and 5 to allow these domains to bind dimeric IgA [15]. Interestingly, domains 3 and 4 have also been found to play an important role in the binding of the SpsA surface protein of *Streptococcus pneumonia*, allowing this bacterium to hijack the receptor and attach to host epithelial cells [14]. Domain 5 of the pIgR forms a disulfide bond between the receptor and the Fc region of dIgA and acts as a bridge between the extracellular domain and the transmembrane domain of the receptor [14]. Following the binding of dIgA to the pIgR, the complex is internalized into a mucosal epithelial cell through the process of endocytosis and transcytosed to the apical side of the cell in a vesicle [14]. A 22-amino cleavage site is recognized by a pIgR-specific proteinase that cleaves domain 5 of the pIgR receptor, though the identity of this proteinase remains unclear [16]. The cleaved portion of the pIgR bound to IgA is termed the secretory component (SC) and consists of domain 1 through 5 of the original receptor [15]. The domains of the SC fold back on each other and form a condensed triangular shape with a solvent accessible gap in the middle [17]. The SC also contains seven N-linked glycosylation attachment sites for carbohydrate chains involved in mediating interactions with bacteria and host cell lectins [17]. The cleaving of the dIgA-SC complex leads to the creation of the final form of IgA termed secretory IgA (sIgA) [15]. The concluding cytoplasmic tail region of pIgR contains signals that are crucial for the transcytosis of IgA across mucosal epithelial cells and for ensuring the receptor is not targeted for lysosomal degradation [14]. The cleaved sIgA complex is released at the apical surface into luminal secretions, such as mucus, where it remains localized and carries out its immunoprotective functions [15].

### 2.2. IgA Function and Mechanism of Action at Mucosal Surfaces

When a mature B cell encounters an antigen in the peripheral lymphoid tissues, it becomes activated and induces the expression of activation-induced cytidine deaminase (AID), which is an enzyme vital in the class-switching recombination (CSR) process of antibody-producing genes [18]. In the instance of IgA expression, the AID enzyme initiates CSR from the Cµ gene that encodes IgM to the Cα gene, which encodes IgA [18]. This process occurs through the cytokine-mediated activation of the Iα promotor located upstream of the Cα gene, leading to the production of a non-coding mRNA transcript called Iα- Cα, which binds to a switch region or S-region upstream of Cα [18]. The binding of the Iα-Cα mRNA to the S-region opens its chromatin structure, making cysteine residues accessible to the AID, which generates double-stranded breaks in the DNA [19]. The non-homologous end joining (NEHJ) DNA repair machinery joins the double stranded breaks and loop out a portion of the DNA in the S-region [19]. As a result, an antibody variable region that determines antigen specificity becomes linked to the Cα gene, which encodes a constant antibody region. This fusion event creates a sequence that encodes the IgA protein [19].

**Figure 2:**
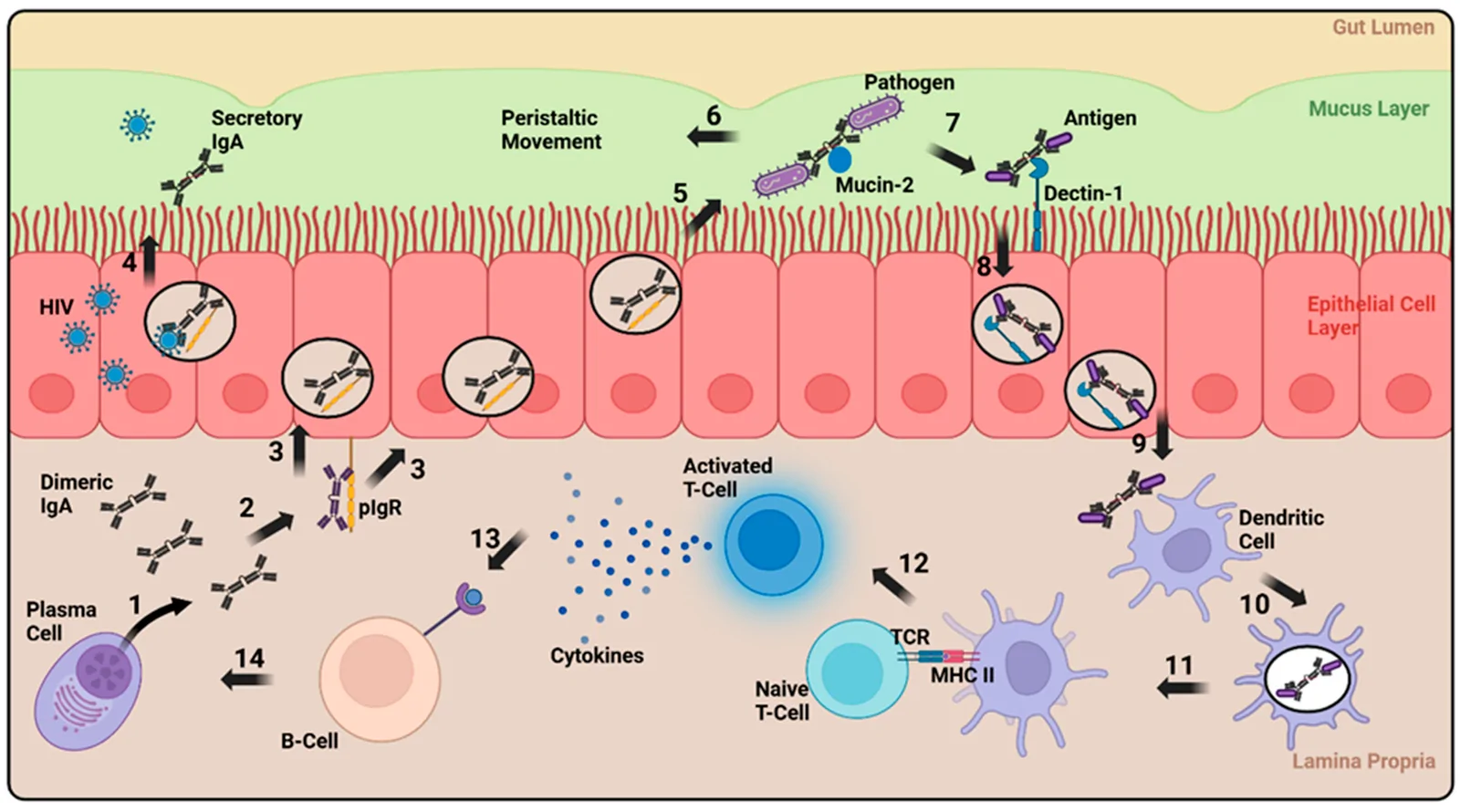
Overview of sIgA production and mechanisms of action at mucosal surfaces. (1) dIgA is produced from plasma cells in the lamina propria. (2) dIgA binds to pIgR on the basolateral surface of mucosal epithelial cells. (3) The pIgR-dIgA complex undergoes transcytosis across mucosal epithelial cells. (4) Intracellular dIgA can bind pathogens such as HIV and release them at the apical surface of epithelial cells for clearance. (5) The pIgR undergoes cleavage at the apical surface of epithelial cells releasing sIgA which can anchor itself at mucosal surfaces using mucin-2 and crosslink pathogens. (6) Pathogens crosslinked by sIgA can be cleared by peristaltic movement of mucus. (7) sIgA can bind antigens and interact with epithelial cell surface receptors such as Dectin-1. (8) The antigen-sIgA complex undergoes reverse transcytosis to the basolateral side of mucosal epithelial cells. (9) The antigen-sIgA complex encounters dendritic cells in the lamina propria. (10) The dendritic cell uptakes the antigen-sIgA complex. (11) The major histocompatibility complex class II (MHC II) of the dendritic cell presents the antigen to the T-cell receptor (TCR) of a naive T cell. (12) The naïve T cell differentiates into a CD4^+^ T-helper cell releasing cytokines. (13) The cytokines released by T cells bind cytokine receptors on B cells. (14) The B cells undergo differentiation into dIgA producing plasma cells. Created in BioRender. Wootton, S. (2026) https://BioRender.com/cyhgs6v (accessed on 1 July 2026).

The production and secretion of sIgA at mucosal surfaces in response to antigens allows the antibody to perform a variety of effector functions, with the most notable being immune exclusion [20]. The immune exclusion process mediated by sIgA prevents pathogens and foreign antigens from adhering to, penetrating, and infecting mucosal epithelial surfaces [20]. The first step of immune exclusion involves the agglutination or crosslinking of invading pathogens forming clumps of bacteria in the mucus layer of the intestinal mucosa (see Figure 2) [21].

Due to its multivalent structure, sIgA can agglutinate invading pathogens containing polyvalent surface antigens or many copies of the same epitope on their surface [21]. This allows a single sIgA to bind multiple pathogens such as bacteria at the same time, facilitating the crosslinking process [21]. Remarkably, research has shown that agglutination does not normally harm bacteria, with bacterial growth rates remaining unchanged after crosslinking [21]. However, studies of the binding of the O-antigen of *S. typhimurium* to sIgA found that this event leads to a degree of physical distortion of the outer membrane and changes in gene expression detrimental to the bacterium [21]. Consequently, the crosslinking of *S. typhimurium* with sIgA antibodies targeted to the flagella of the bacterium did not lead to any structural changes in its membrane [21]. This shows that structural effects and gene expression changes in bacteria as a result of agglutination are likely dependent on the epitope recognized by the sIgA [21].

The second step of immune exclusion is the physical entrapment of pathogens by sIgA in the mucus layers present at mucosal surfaces [22]. Before sIgA agglutinates bacteria, it binds to the proteins present in the mucus layers most notably mucin-2 (Muc2) and this process is enhanced by the presence of the SC, which contains seven carbohydrate side chains that anchor the antibody at mucosal surfaces [22,23]. This process allows IgA to trap pathogens in mucus layers, leading to them being cleared by the natural process of mucus turnover and peristaltic movement at mucosal surfaces [22].

In addition to immune exclusion, IgA can also mediate a number of additional effector functions through interactions with a variety of host-cell receptors on different cell types [24]. IgA interacts with the FcαRI (CD89) receptor expressed on neutrophils and other myeloid cells, triggering effector functions such as phagocytosis, degranulation, reactive oxygen species production, and cytokine release that contribute to pathogen clearance [24]. Indeed, the binding of IgA to the FcαRI receptor on neutrophils leads to the production of the LTB4 chemoattractant through the ITAM signaling pathway, which attracts additional immune cells resulting in an amplification of this process [24]. After the binding of IgA to FcαRI on neutrophils, this leads to pathogens being phagocytosed and eliminated by an NADPH oxidase respiratory burst in the neutrophil interior [25]. It is important to note that the partial binding of IgA to the FcαRI can cause incomplete phosphorylation of the receptor, leading to the downregulation of neutrophil recruitment, in which case, an inhibitory ITAM signaling pathway is engaged to reduce inflammation [26]. IgA can also bind to the Dectin-1 receptor located on the apical surface of M cells in Peyer’s patches of the intestines allowing the reverse transcytosis of IgA-antigen complexes to occur [27]. Specifically, antigen-bound sIgA2 binds to Dectin-1 through its glycosylated cα1 domain allowing the endocytosis of the IgA-antigen complex from the apical surface of mucosal epithelial cells to the basolateral surface [27]. The IgA-antigen complex is then released into gut associated lymphoid tissues at the basolateral surface where the complex can be taken up and eliminated by resident dendritic cells (see Figure 2) [27].

In contrast to the reverse transcytosis process, IgA has also been found to harbor an immune excretion process specific to pathogens that replicate in the lamina propria [28]. In addition to mediating immune exclusion at mucosal surfaces, dIgA can facilitate the removal of pathogens that have already crossed the epithelial barrier. For example, dIgA has been shown to bind HIV virions within the lamina propria and form immune complexes that engage pIgR on the basolateral surface of epithelial cells. These complexes are subsequently transcytosed to the apical surface and released into the lumen, thereby removing virus from subepithelial tissues and limiting access to susceptible CD4^+^ T cells and other target cells (see Figure 2) [28]. IgA also plays a role in the intracellular neutralization of bacteria and viruses [29]. When the IgA-pIgR complex is undergoing transcytoses across epithelial cells, dIgA can colocalize with endocytosed lipopolysaccharide (LPS), which is a toxic outer membrane component of bacteria [29]. This colocalization allows IgA to inhibit the ability of LPS to trigger an NF-κB mediated cytokine response limiting inflammation and maintaining homeostasis in epithelial cells [29]. Unlike immune excretion of luminal antigens such as LPS, transcytosing dIgA specific for the Sendai virus hemagglutinin–neuraminidase (HN) protein mediates intracellular neutralization by intercepting viral components within infected epithelial cells during pIgR-mediated transport [30]. The pIgR-bound dIgA enters the same protein trafficking pathways as the HN causing colocalization to occur and preventing the proper assembly and budding of the Sendai virus at the apical surface of epithelial cells [30].

Maternal sIgA plays a central role in the establishment of newborn commensal microbiota following exposure to the environment and maternal microbes [21]. Newborns acquire maternal sIgA through breast milk, with the antibody guiding the establishment of the newborn microbiota through binding commensal bacteria and facilitating controlled colonization [21]. The colonization of the newborn microbiota in turn leads to the maturation of gut-associated lymphoid tissue (GALT) with the limited production of IgA against commensal microbiota to limit redundant inflammation [21]. A deficiency in the production of IgA in humans characterized as selective IgA deficiency (sIgAd) leads to a reduction in the diversity and an imbalance in proportions of microbes present in the microbiota [31]. The clinical signs of this disease are often masked by the compensatory production of IgM which also binds and keeps commensal bacteria in check [31]. However, individuals with sIgAd still possess a shifted microbiota composition with IgM not being able to fully compensate for the absence in production of IgA or its specialized role in maintaining homeostasis [31].

### 2.3. Advantages of sIgA over IgG for Mucosal Pathogens

Although both sIgA and IgG play roles in defending against mucosal pathogens, sIgA is uniquely adapted to mucosal environments, whereas IgG is largely involved in whole-body immunity (see Table 1).

As mentioned before, dIgA is produced locally by plasma cells in the lamina propria, allowing the transcytosis and secretion of sIgA directly at mucosal surfaces where it can induce immune exclusion before pathogens are able to invade. In contrast, IgG antibodies are primarily produced in the serum by the plasma cells of the spleen with the function of encountering pathogens that have already invaded epithelial tissues and entered the bloodstream [32]. It is important to note that IgG can undergo transcytosis from the lamina propria to the surface of mucosal epithelial cells using the neonatal Fc receptor (FcRn) [33]. Experiments have shown that IgG can then bind antigens present at mucosal surfaces and move back through epithelial cells into the lamina propria to encounter dendritic cells for processing [33]. Though IgG is present in mucosal secretions, it is released in significantly smaller concentrations than IgA and is only reactive toward infection rather than preventive [33]. As discussed previously, dIgA is able neutralize a variety of pathogens intracellularly when being transported by pIgR to the apical surface of epithelial cells compared to IgG, which can only perform this function for specific pathogens during transport by FcRn [34]. IgG has only been loosely characterized in the literature to perform intracellular neutralization against the VP6 capsid protein of rotavirus in which the Fc region of IgG is able to bind TRIM21-bound pathogens and target them for degradation [34]. In contrast, IgA has been well characterized to be involved in the intracellular neutralization of multiple viruses including Sendai virus, influenza virus and HIV [30,35]. The broad intracellular neutralizing ability of IgA to inhibit viral replication provides it with an advantage over IgG, which is largely restricted to extracellular effector functions [32]. The immune exclusion process used by IgA elicits a considerably reduced pro-inflammatory response in comparison to the IgG effector function of binding Fcγ-receptors on immune cell surfaces and promoting phagocytosis [36]. While the IgG pro-inflammatory response is vital in the clearing of pathogens, this same response can cause damage to tissues when misdirected towards the body’s own cells in the case of autoimmune diseases [32]. Specifically, IgG can mistakenly bind to self-receptors and form immune complexes that can lead to the activation of immune cells that attack the body’s own tissues [37]. These immune complexes can additionally activate complement signaling cascades, including the classical, alternative and lectin pathways, leading to additional damaging inflammatory responses in the body [37]. Since IgA primarily uses the process of immune exclusion to neutralize pathogens in the mucosa, immune cell-activating pathways are not induced at levels comparable to IgG, meaning the risk of collateral tissue damage is reduced [38]. Research has shown that due to its distinct glycosylation profile, IgA does not efficiently activate the classical complement pathway like IgG and instead engages the lectin pathway, leading to weaker overall complement activation [39]. As referenced earlier, IgA is crucial in maintaining mucosal homeostasis by preventing excessive inflammatory responses against commensal microbes compared to IgG, which can elicit elevated immune responses [31,40]. In a study related to ulcerative colitis, a murine model was created that mimicked the effects of the disease, notably the increase in production of IgG from B cells located in the gut normally dominated by IgA [40]. This led to an elevated number of commensal microbes in the gut being coated with IgG, with these complexes activating harmful immune responses that would normally not be induced by IgA opsonization [40]. It should be noted that the increased production of and binding of IgA to commensal bacteria has also been observed to induce harmful immune responses in a small subset of ulcerative colitis patients, though the correlation is drastically reduced compared to IgG [41].

**Table 1:** Comparative advantages of sIgA versus IgG at mucosal surfaces.

Feature	sIgA	IgG	Advantage	Reference
Site of production	Produced by plasma cells in the lamina propria, enabling immediate secretion at mucosal surfaces	Primarily produced by plasma cells in the spleen and bone marrow, reaching mucosal surfaces by serum	sIgA can neutralize pathogens before they breach the epithelium	[32]
Immune exclusion	Traps pathogens at mucosal surfaces without triggering strong inflammatory responses	Functions after pathogen invasion promoting phagocytosis and inflammation	sIgA neutralizes pathogens while minimizing inflammatory responses which can cause tissue damage	[36,37]
Complement activation	Limited to the lectin pathway causing minimal activation	Activates classical, alternative and lectin pathways leading to potent inflammation	sIgA reduces excessive complement mediated inflammation thereby preventing collateral tissue damage	[37,38]
Interaction with commensal microbes	Prevents overactivation of immune system against commensal microbes	Can induce harmful immune responses when coating commensal microbes	sIgA supports mucosal homeostasis, limiting harmful immune responses	[40,41]
Mucosal anchoring	Binds to multiple mucin proteins at mucosal surfaces allowing long-term retention	Possesses limited ability to bind mucin proteins diffusing more freely at mucosal surfaces	sIgA remains localized at mucosal surfaces for extended periods of time allowing enhanced pathogen neutralization	[42,43]
Protease resistance	Resistant to degradation by proteases due to SC	More susceptible to protease degradation	sIgA maintains function in protease rich mucosal environments extending protective effects	[44,45]
Pathogen crosslinking	Dimeric structure increases its ability to crosslink multiple pathogens simultaneously	Monomeric structure limits crosslinking activity	sIgA more effectively neutralizes pathogens by immune exclusion using multivalent interactions	[46,47]
Antibody-dependent enhancement (ADE)	Low risk of ADE when non-neutralizing	High risk of ADE when non-neutralizing	sIgA reduces the risk of ADE, limiting enhanced infection	[48]

As was highlighted earlier, IgA can interact with proteins such as mucin-2 at mucosal surfaces using the SC to anchor itself in the mucosa in contrast to IgG, which largely diffuses through the mucosa [42]. IgG has been found to anchor itself in the mucosa through the binding of mucin proteins using the IgG Fc-binding protein (FcGBP), though this interaction is limited in its ability to prevent the diffusion of IgG [42,43]. Conversely, the interaction of sIgA with mucin proteins is strong, allowing the anchoring of the antibody at mucosal surfaces for longer periods of time with little diffusion [43]. The enhanced ability of sIgA to remain anchored in the mucosa over IgG provides the advantage of increased contact and neutralization of pathogens at mucosal surfaces [43]. Although sIgA has a shorter half-life in serum than IgG, the structural SC provides IgA with increased resistance to proteases at mucosal surfaces as mentioned earlier [44,45]. In an experiment in which sIgA, dIgA and IgG were digested with purified Proteus Mirabilis protease, it was observed that sIgA was degraded at a slower rate than IgG and dIgA, showing the importance of the SC component in protease resistance [44].

The dimeric structure of IgA provides it with an increased ability to bind and crosslink pathogens over IgG, which only exists in a monomeric structure [46]. A study was conducted in which the avidity and affinity of monomeric IgA, dIgA and monomeric IgG to bind to the M2 protein of influenza was evaluated [47]. It was found that dIgA more effectively inhibited viral plaque formation than monomeric IgA and IgG since it was able to bind a larger subset of M2 proteins [47]. However, there was no significant difference in the affinity of dIgA to bind M2 compared to monomeric IgA and IgG, meaning the polymeric nature of IgA only increases its avidity for binding antigens and not the strength in which it can bind antigens [47]. Additionally, IgA is less likely to induce antibody-dependent enhancement (ADE) compared to IgG in which non-neutralizing antibodies can bind pathogens and facilitate their increased uptake into host cells [48]. Unlike IgG, which can promote FcγR-mediated uptake of virus–antibody complexes and facilitate ADE when antibodies are non-neutralizing, IgA engages FcαRI in a way that is less efficient at promoting viral entry and is therefore much less likely to drive ADE [48]. Research has found that an IgA version of a dengue virus-reactive antibody was able to interfere with the ability of the IgG version to induce ADE due to competition for antigen-binding sites [49]. These findings indicate that the amount of dengue-specific IgA produced during infection can influence the severity of IgG-mediated ADE and serve as a biomarker for disease susceptibility [49].

## 3. Strategies for sIgA Production and Platforms for Vectorized Expression In Vivo

### 3.1. Challenges with Engineering sIgA Expression

Despite the significant potential of sIgA as a first line of defense at mucosal surfaces, there are many complexities associated with its recombinant expression in vivo [50]. One of the main challenges of expressing sIgA lies in using expression vectors to reproduce the precise stoichiometric co-expression ratios of its components, including the heavy chain, light chain, Jchain and SC that occurs naturally at mucosal surfaces [50]. sIgA is the product of two separate cell types, with the SC being a cleaved product from the pIgR on epithelial cells and the other components originating from plasma cells in the lamina propria [51]. This means that each of the components of dIgA need to be expressed at precise ratios to form complexes with the pIgR on epithelial cell surfaces for proper assembly to occur [51]. sIgA was notably produced in a dIgA-expressing mammalian cell line in 1997 with the transfection of an SC expression vector proving the viability of recombinant sIgA production in vitro [52]. However, of the IgA components produced in single cells, it was found that only a fraction of the components assembled into sIgA with many forming monomeric IgA or dIgA [52]. It is thought that this anomaly could be a result of suboptimal expression levels of the dIgA components with SC and that using promotors of varying strengths or increasing gene copy numbers could improve sIgA expression [53]. The formation of sIgA is also dependent on proper post-translational modifications of its components including O- and N-linked glycosylation, with these modifications being host cell-dependent [54]. A study looking at the difference in IgA glycosylation after expression in plant and HEK293 cells found significant differences in glycosylation patterns, with the glycosylation of IgA in HEK293 cells providing improved stability [55]. These findings are significant since the glycosylation patterns of IgA could be altered when being expressed in vivo depending on the location of expression, with these alterations affecting proper IgA formation and stability [55]. Differences in glycosylation can also influence IgA interactions, with the J-chain and pIgR adding an additional hurdle to in vivo expression [54]. Another challenge associated with sIgA expression in vivo is the tissue-specific targeting of dIgA expression at mucosal epithelial cells where it can interact with the pIgR and be expressed at mucosal surfaces. The expression of dIgA mainly occurs in the plasma cells of the lamina propria adjacent to mucosal epithelial cells in the gastrointestinal tract and respiratory tract. This means that expression of plasmids encoding the dIgA components must be delivered to mucosal epithelial cells in a highly specific manner using transport vectors such as viruses or nanoparticles. Previous work has shown that the engineered adeno-associated virus capsid AAV6.2FF has strong tropism for lung epithelial cells, particularly alveolar type II cells, and was able to efficiently expression of IgG antibodies in the lungs of murine models [56]. These findings support the feasibility of tissue-targeted delivery using vectors such as AAV to achieve sustained antibody expression [56]. The use of transport vectors for in vivo expression of recombinant antibodies can, however, be complicated by host immune responses notably the activation of complement and cytokine signaling pathways [57]. Studies have shown that the administration of AAV vectors can lead to the activation of host immune responses against the viral capsid proteins along with therapeutic transgenes [57]. An ongoing area of research is focused on the removal of the major pathogen-associated molecular patterns (PAMPS) from AAV vectors, though it is unlikely that this will completely eliminate the stimulation of host immune responses [58]. Evidence shows that lipid nanoparticles specifically the ionizable lipids also lead to the activation of cytokine signaling pathways, showing the widespread issues of immune responses towards expression vectors [59]. In addition, the use of gamma globulin therapy, in which IgA-rich preparations derived from donor plasma are given to IgA-deficient patients, has been shown to induce anti-drug antibody (ADA) responses [60]. Specifically, IgG anti-IgA antibodies that were already present in the IgA-deficient patients reacted to the increased levels of IgA, causing complement activation in a subset of patients [60]. Although ADA responses to administered IgA have been reported, it is not yet clear whether in vivo IgA expression will elicit comparable responses, underscoring the need to evaluate and mitigate potential immunogenicity [60]. AAV-mediated expression of broadly neutralizing IgG antibodies against HIV has been shown to induce ADA responses in non-human primates and humans, providing evidence that this phenomenon could occur for AAV-expressed IgA as well [61].

### 3.2. Overview of Platforms for the Expression of IgG and sIgA In Vivo

AAV-based gene delivery has emerged as a leading strategy for the long-term in vivo expression of IgG antibodies [62]. AAV gene therapy vectors have an icosahedral structure composed of three viral capsid proteins along with a genome capable of encoding a maximum of 4.7 kb of DNA flanked by inverted terminal repeats (ITRs), with all other components of the original viral genome removed making the vectors replication deficient [63]. At least thirteen distinct natural serotypes of AAV have been identified, each with characteristic tissue tropisms [64,65]. Following infection of host cells, AAV delivers its genome to the nucleus where it forms stable, transcriptionally active episomes that express target genes without integration into the host genome [63]. AAV-mediated gene delivery has been extensively investigated for the in vivo expression of IgG monoclonal antibodies targeting a wide range of viral, bacterial, and parasitic pathogens. Across numerous pre-clinical studies, AAV-mAb expression has consistently demonstrated protective efficacy and the capacity for durable, long-term IgG expression, supporting its potential as an alternative to traditional vaccination or passive immunization approaches. While an extensive body of work spans multiple infectious disease models, representative studies have shown that AAV-directed expression of antiviral IgG can confer complete protection in small animals and sustain circulating antibody levels for the lifespan of the host [66,67,68,69]. For example, AAV-mediated delivery of IgG against filoviruses such as Marburg and Ebola has achieved complete protection in mice and guinea pigs, with antibody expression maintained long term [66,67,68,69,70]. Similarly, translation to large-animal models has demonstrated the scalability and durability of this approach; AAV6.2FF-mediated IgG expression in lambs resulted in sustained antibody production for over 1100 days [71]. Notably, although anti-drug antibodies (ADAs) against the expressed IgG and anti-capsid immune responses have been observed, these responses were low and remained stable over time in this model, suggesting that immunogenicity may not preclude prolonged transgene expression in all settings.

Replication-deficient adenoviral vectors, which encompass multiple serotypes with distinct tissue tropisms, represent an in vivo expression system for IgG antibodies [72]. Adenoviral vector icosahedral capsids are approximately 100 nm in diameter and can package up to 36 kb of DNA, with the viral genome being maintained as an episome similar to AAV [73]. There are three generations of adenoviral vector systems with the most widely used being the third-generation system in which the genome has been mostly depleted of adenoviral DNA with the exception of the ITRs and packaging signals required for vector production [72]. An adenoviral vector system was used for the expression of recombinant IgG antibodies against the TcdA and TcdB toxins produced by clostridium difficile and fully protected mice against lethal challenge by the bacterium, with strong antibody expression lasting for over a month [74]. Despite the success of this study, use of these systems has been complicated by systemic inflammatory responses and modest duration of IgG expression [75].

Beyond viral platforms, lipid nanoparticle (LNP) delivery systems, which are composed of four distinct lipid types, offer an alternative method for in vivo expression of IgG antibodies [76]. Current LNPs consist of ionizable lipids important in the encapsulation of nucleic acids and endosomal uptake, helper lipids which support LNP stability, cholesterol that limits nonspecific protein binding to the LNP surface, limiting inflammatory responses, PEG-lipids that give LNPs consistent size, and prevent aggregation and mRNA of variable sizes encoding proteins of interest [76]. A significant challenge of using LNPs for tissue-specific expression of antibodies is the fact that they are naturally taken up by cells in the liver and that targeted expression is difficult [77]. However, recent advances incorporating selective organ targeting (SORT) lipids into LNPs have enabled tissue-specific expression, with positively charged SORT lipids directing delivery to the lungs and negatively charged SORT lipids targeting the spleen [77]. LNPs have been shown to induce sustained expression of an anti-SARS-CoV-2 human IgG mAb in mice, providing protection against lethal challenge for over 63 days [78]. Despite the advent of SORT methods, targeted delivery of LNPs to tissues remains a significant barrier for gene delivery [77].

Regarding in vivo expression of sIgA, there have currently been two methods used including lentiviral vector-mediated gene delivery and LNP-mediated mRNA delivery. Lentiviral vectors have a ~10 kb packaging capacity [79]. The third-generation lentiviral system, which is widely used due to its improved safety profile, employs four plasmids: one encoding gag/pol, one encoding the envelope protein for cell-specific targeting, one carrying the transgene under a promoter, and one encoding rev for nuclear export. These systems are typically designed as self-inactivating (SIN) through deletion of the U3 region in the 3′ long terminal repeat (LTR) [79]. The co-transfection of these plasmids in vitro leads to the production of lentiviral vectors that can target and integrate their transgenes into host cell genomes for prolonged expression [80]. Lentiviral vectors containing anti-HIV sIgA transgenes were used for the ex vivo transduction of hematopoietic stem cells, after which these stem cells were transplanted into the bone marrow of humanized mice [81]. It was found that the anti-HIV sIgA was expressed in plasma cells in lymphoid tissues preventing their depletion following lethal HIV challenge [81]. This study demonstrates an indirect approach in which sIgA is expressed in vivo using stem cell therapy, in contrast to the direct delivery methods used for IgG expression by adenoviral and AAV vectors [81]. Despite the success of lentiviral vectors in expressing IgA in vivo, the risk of transgene integration near proto-oncogenes leading to cancer remains a major hurdle to the widespread use of these systems along with the challenges of stem cell therapy [82]. A recently conducted study investigated the use of LNPs for the delivery and expression of sIgA mAbs into mucosal secretions for protection against pseudomonas bacteria [83]. Mice were IV-injected with 1 mg/kg of LNPs containing mRNA encoding the IgA heavy chain, light chain, and J-chain alongside the injection of mice with 5 mg/kg of recombinant IgA produced in cell culture and 4.5 mg/kg of polyclonal IgA isolated from serum [83]. It was found that the recombinant IgA had the shortest serum half-life of 0.64 days with expression dropping of after 2 days, followed by serum-derived IgA with a half-life of 0.93 days with expression dropping of after ~12 days, and mRNA-derived IgA having a half-life of 1.63 days with expression dropping off after ~16 days [83]. The authors speculated that the reduced half-life of serum- and cell culture-derived IgA may be due to differences in glycosylation patterns arising from expression in different murine cell types [83]. Additionally, it was found that the expression of mRNA-derived IgA protected mice against lethal challenge, with *S. enterica* in the intestines and *Pseudomonas aeruginosa* in the lungs [83]. The conclusions of this work show the feasibility of the production of recombinant sIgA at mucosal surfaces in vivo and the importance of host glycosylation patterns for the stability of expressed antibodies. A major limitation of this study is that LNP-mediated IgA expression peaked 24 h after administration before gradually declining until day 16, paving the way for longer term expression using vectors such as AAV [83].

### 3.3. Proposed Design Considerations for the Correct Assembly of sIgA In Vivo

**Figure 3:**
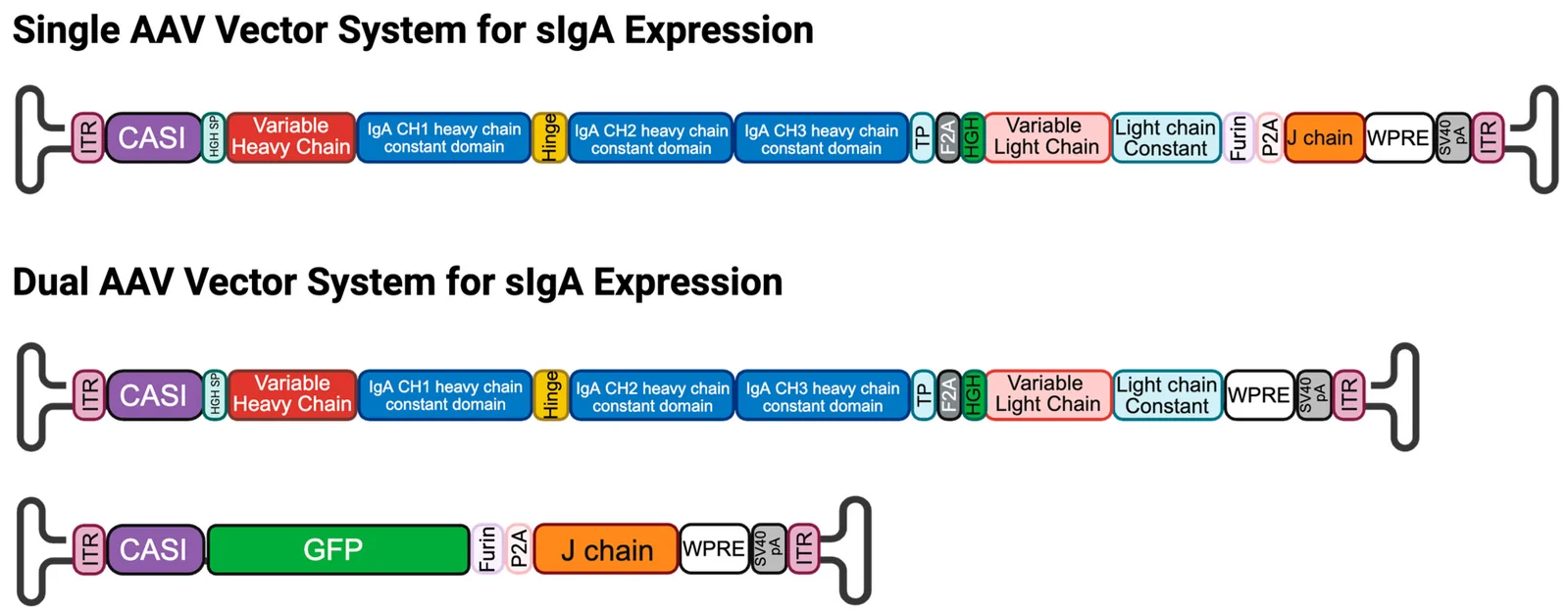
Overview of dual and single AAV vector systems and mechanisms of action. Potential components of IgA expressing AAV vector genomes (vg) for single and dual vector systems. Created in BioRender. Wootton, S. (2026) https://BioRender.com/iqyzepk (accessed on 1 July 2026).

The correct assembly of sIgA in vivo remains a significant challenge requiring the careful consideration of plasmid design, expression balance of components, and vector delivery systems [50]. The design considerations discussed in this section represent a proposed framework for the efficient production of sIgA in vivo derived from an analysis of the current literature and have not yet been combined and experimentally tested as an integrated platform to our knowledge. For the proper in vivo expression of sIgA to occur, the heavy chain, light chain, and J-chain must be expressed for proper assembly to occur as mentioned previously [50]. If AAV is to be used, the genes encoding all four components of sIgA would not fit into a single plasmid, but it would be possible to express the IgA-J chain components using a polycistronic system linking each gene with 2A self-cleaving peptides [84]. The 2A self-cleaving peptides originate from viral genomes and induce ribosome skipping during translation producing two separate proteins from a single mRNA [84]. An advantage of using 2A self-cleaving peptides over other strategies for multi gene co-expression is that they are only 18–22 amino acids in length, meaning they add minimal size to plasmids, and the proteins are expressed in equimolar amounts provided ribosomal skipping is 100% efficient [85]. Many different 2A peptides exist, including P2A, T2A, F2A, and E2A with each having different cleavage efficiencies and effects on downstream expression [85]. Experiments have demonstrated that the T2A led to the highest expression of a second gene in a bicistronic system while the combination of using T2A first, P2A second, and E2A third led to the highest expression of the fourth gene in a quad-cistronic system [85]. For the expression of sIgA from a single plasmid, a tricistronic system could be used in which the heavy and light chains are separated by T2A, the J-chain separated by P2A (see Figure 3) [85].

In this proposed design, the components of this single plasmid could be expressed under a single CASI promotor and packaged in a single viral vector such as AAV [86]. The CASI promotor consists of cytomegalovirus enhancer (CMV) and a chicken beta actin promotor and has been shown to elicit strong expression of proteins from a range of tissues, including lung and muscle, following AAV transduction [86]. A previous study has used a method similar to that above for the expression of IgG in which a bicistronic system was used for the expression of the heavy and light chains from the same AAV vector [87]. Despite the advantages of using a single polycistronic system for sIgA expression, it has been demonstrated that the translation of genes further from the promoter decreases due to ribosome drop-off [85]. As a result, the genes placed at the end of the proposed polycistronic system for IgA expression could be drastically underexpressed [88]. In light of this, the components for IgA expression could be separated with the heavy and light chain genes being placed on a plasmid, creating a bicistronic system and the J-chain being placed on an separate plasmid (see Figure 3) [89]. Each of these plasmids could be packaged into two separate vectors such as AAV and co-administered in vivo for the expression of IgA, mitigating the effects of ribosome drop-off [89]. Previous research has shown that using dual AAV systems provided stronger expression of IgG antibodies in fibroblast cells but showed no enhanced expression in other cell types such as HEK293T cells compared to a single vector approach [89]. The use of dual AAV vector systems provides an additional advantage of allowing transgenes to be placed under different promotors and enhancer elements, thus allowing more precise stoichiometric expression [90]. However, a notable disadvantage of dual AAV vector systems is decreased transduction in certain tissues thought to be due to receptor competition [90]. In contrast to using single promotor bicistronic systems, dual promotor systems could be used, allowing precise stoichiometric expression of sIgA components without ribosome drop-off [91]. Dual promotor systems have been successful in the production of IgG antibodies from a single plasmid, though interference of transgene expression due to the close proximity of promotors remains a significant challenge [91,92]. In addition to using the CASI promotor for expression of IgA, the woodchuck hepatitis virus post-transcriptional enhancer (WPRE) could also be included in the plasmid design as this was shown to increase IgG expression 2-fold [93]. A furin cleavage site upstream of the 2A sequences could also be utilized for the removal of 2A-derived residues at the end of translated proteins, allowing more efficient folding to occur [94]. The additional use of linker sequences such as GSG between 2A sequences and transgenes has been found to increase the efficiency of protein folding through limiting steric hindrance [94]. Since sIgA is a secreted protein, a human growth hormone (HGH) signaling peptide could be included in the plasmid design to enhance the secretion of the heavy and light chains [62]. Specifically, an HGH sequence could be placed in front of the IgA heavy chain and a different HGH DNA sequence encoding the same protein be placed in front of the light chain to mitigate recombination-mediated deletion [62]. A polyadenylation signal such as the SV40 polyA is required to direct proper cleavage and polyadenylation of the mRNA, resulting in the addition of a poly-A tail [95]. As mentioned earlier, the expression of IgA can induce ADA responses that could be dampened through incorporating miRNA-binding sites in the plasmid design [96]. The mir-142-3p miRNA is naturally expressed by antigen-presenting cells (APCs) and the incorporation of binding sites in plasmids allows for transgene suppression [96]. The inhibition of transgene expression in APCs is favorable as this prevents the presentation of expressed proteins to T cells triggering an immune response [96]. Another approach to combat ADA responses is the inclusion of a PD-L1-expressing gene in the plasmid. PD-L1 is an immune checkpoint ligand that binds the PD-1 receptor on activated T cells, leading to inhibition of T-cell activation and reduced cytokine production [97]. A previous study found that the co-expression of an immunogenic luciferase transgene with PD-L1 increased transgene expression over time compared to non-PD-L1 expressing controls [97,98]. Existing literature has shown that the overexpression of recombinant antibodies in Chinese hamster ovary (CHO) cells does not trigger an upregulation of the chaperone proteins disulfide isomerase (PDI) or heavy chain-binding protein (BiP), both of which are required for proper antibody folding [99]. Notably, co-transfection of plasmids encoding PDI and BiP into antibody-producing CHO cells increased antibody folding efficiency and secretion, highlighting an additional plasmid-design consideration for sIgA [99]. Furthermore, studies have shown that the production of recombinant antibodies in CHO cells led to incomplete glycosylation due to these cells not expressing all the necessary glycosyltransferase enzymes [100]. To combat this, glycosyltransferase enzyme genes were engineered into the CHO cell genome, allowing the overexpression of these enzymes and the proper glycosylation of recombinant antibodies to occur [100]. This approach could be applied to enhance the stability of sIgA in vivo by the addition of glycosyltransferase enzyme genes in the plasmid design or on a separate plasmid in a dual vector system for proper glycosylation [100]. It is important to note that all sequences incorporated in the plasmid design mentioned above could be codon-optimized for the species in which protein expression will occur to improve translation efficiency [101]. Codon optimization is completed through the adaptation of nucleotide sequences to encode a host species with the most abundant tRNA and can increase transgene expression 44-fold [101]. Overall, incorporating the plasmid design and vector delivery strategies above could provide a potential route for the production of sIgA in vivo [50].

### 3.4. Potential Routes of Administration for VIP-Mediated sIgA Expression

The effective delivery of recombinant antibodies to desired tissues is dependent on the route of administration, which can determine if antibodies are expressed locally in tissues or systemically throughout the body. The routes of administration discussed in this section are proposed based on their potential to facilitate sIgA expression at mucosal surfaces and have not been experimentally validated in animal models to our knowledge. Intranasal administration is minimally invasive and used for targeted delivery of viral vectors to the lungs, with this method being well suited for vector-mediated expression of sIgA at respiratory mucosal surfaces [102]. However, IN administration often results in reduced transgene delivery to distal surfaces of the lungs compared to more invasive routes of administration [102]. The intratracheal (IT) administration is a more invasive method used for the delivery of viral vectors to the lungs requiring the surgical exposure of the trachea for injection [103]. Despite the invasiveness of this procedure, it provides direct access to the lungs, allowing enhanced transgene expression within the lower airways making it ideal for sIgA expression in vivo [103]. Intramuscular (IM) administration is used for the delivery of transport vectors to muscle cells and is often performed in the gastrocnemius of mice [104]. IM administration also leads to systemic transgene expression throughout the body, in addition to muscle cells, and can cause elevated immune responses, meaning that it is likely not the optimal route to express sIgA, which must be expressed at mucosal surfaces to be most effective [104]. Intraperitoneal (IP) administration involves the delivery of viral vectors through the abdomen of mice into the peritoneal cavity [105]. Although targeted antibody expression is possible using IP administration, this method is often used for the systemic expression of transgenes in organs lining the peritoneal cavity rather than the precise mucosal expression needed for sIgA [106]. Oral administration of viral vectors is completed by oral gavage in which vector preparations are delivered through feeding tubes to the stomach of mice for targeting of the intestines [107]. Despite the feasibility of recombinant protein expression in the GI tract, oral administration is complicated by the highly acidic environment and the rapid turnover rate of the intestinal epithelium [108]. The vectored-mediated delivery of sIgA to intestinal epithelial cells could provide a viable approach for achieving expression at intestinal mucosal surfaces, though expression could be short-lived due to the harsh environment [108]. Intraparenchymal administration is completed by the surgical exposure of the liver in mice and the subsequent injection of vector for targeted delivery to hepatocytes and systemic expression [109]. Though elevated systemic expression of recombinant proteins in the lungs is observed, this route of administration can activate robust inflammatory responses due to the presence of Kupffer cells [109]. The systemic nature of the intraparenchymal administration in regard to lung expression could provide an alternative route for sIgA expression at mucosal surfaces, though immune cell activation could present a limitation [110]. Intravenous (IV) administration is completed by the injection of vector doses in the lateral tail vein of mice, allowing direct delivery to the bloodstream for systemic expression in organs, including the lungs [111]. A disadvantage of IV administration is that achieving high transgene expression in extra-hepatic organs such as the lungs often necessitates high vector doses, elevating the likelihood of liver toxicity [112]. The systemic delivery and expression of sIgA at lung mucosal surfaces could be accomplished by IV administration, but liver toxicity is a potential concern [112]. Since sIgA must be expressed at mucosal surfaces to exert its protective effects, the IN and IT routes of administration remain the most promising for respiratory mucosal expression, although future studies in animal models must be conducted to confirm this conclusion [113].

## 4. Clinical Translation Potential and Future Directions in Vectored IgA Immunoprophylaxis

### 4.1. Lessons from Previous VIP Studies and Relevance to sIgA

Lessons from previous studies focused on the delivery of monoclonal antibodies through VIP provide an important foundation for understanding the challenges and benefits of expressing sIgA in vivo. One of the first major studies focused on VIP involved the AAV-mediated expression of broadly neutralizing mAbs against HIV [62]. This work established that a single intramuscular administration of an AAV vector could drive long-term antibody expression in vivo, eliminating the need for repeated antibody dosing. Importantly, AAV-mediated expression of broadly neutralizing mAbs protected humanized mice from HIV challenge [62]. The implications of this research for IgA suggests that vectored platforms are capable of sustained expression of monoclonal antibodies in vivo. Another important study in this field focused on the comparison of the intramuscular and intranasal routes of administration for AAV encoding respiratory syncytial virus (RSV)-specific IgG antibodies [114]. It was observed that IM administration resulted in high concentrations of antibody expression at mucosal surfaces in the lungs and intestines while IN administration resulted in higher antibody concentrations in the lungs but no expression in the intestines [114]. Additionally, this study included the use of a kill switch system in which the IgG coding sequence was flanked by lox-p sites, allowing the selective excision and repression of the coding sequence by the administration of an AAV encoding Cre recombinase [114]. These results illustrate that administration of AAV directly to the respiratory tract is a viable strategy for driving sIgA expression at the respiratory mucosa, while also highlighting the clinical importance of incorporating a kill switch to terminate antibody expression in the event of adverse effects. Adverse effects from sIgA overexpression could include the altering of microbiome composition through enhanced clearance of targeted microbes, though future studies will be needed to characterize this [31]. A subsequent study explored the correlation between the dose of IgG encoding AAV vectors given to mice and the corresponding transgene expression levels [115]. In particular, three doses of AAV8 were IV administered to mice including 2 × 10^10^, 1 × 10^11^ and 2 × 10^11^ vector genomes per mouse (vg/mouse), with blood and tissues samples collected at multiple time points post injection [115]. The concentrations of vg and IgG were quantified in mouse samples and data revealed that concentrations of both components were highest in the lungs over a period of 25 days after a 1 × 10^11^ vg dose was administered [115]. These results provided valuable insight for sIgA VIP, demonstrating that a 1 × 10^11^ vg dose of AAV may be optimal for the targeted expression of monoclonal antibodies in the lungs of mice, with higher doses providing diminishing returns. An recent study explored AAV-mediated expression of a bispecific IgG antibody against two different *Pseudomonas aeruginosa* target antigens using a bicistronic expression cassette system [116]. Lopes et al. (2024) extended vectored antibody delivery to bacterial infection by engineering an AAV vector to encode the bispecific anti-PcrV and anti-Psl monoclonal antibody, MEDI3902 [116]. The AAV genome was designed to express a single antibody molecule incorporating specificity for both the PcrV component of the type III secretion system and the Psl exopolysaccharide, enabling simultaneous blockade of bacterial virulence and enhanced opsonophagocytic clearance [117]. Following a single intramuscular administration, this AAV construct supported sustained systemic expression of the bispecific antibody in vivo. In a lethal pneumonia model, mice expressing the bispecific mAb exhibited 87.5% survival following challenge with 4.47 × 10^7^ CFU of *Pseudomonas aeruginosa* (PAO1), demonstrating substantial protection, albeit slightly lower than that observed with the monospecific anti-PcrV mAb (100%). Notably, despite lower circulating antibody levels, the bispecific construct was associated with enhanced control of bacterial dissemination to peripheral organs, highlighting the functional advantage of dual-targeting over monospecific approaches [116]. A further study examined the immune responses caused by the expression of anti-simian immunodeficiency virus (SIV) IgG antibodies from AAV8 in non-human primates (NHPs) [118]. Experiments showed that AAV genomes encoding IgG antibodies naturally generated in NHPs in response to SIV infection resulted in significantly lower anti-drug antibody (ADA) responses than AAV-mediated expression of simianized antibodies derived from human sequences [118]. Additional experiments showed that animals with pre-existing immunity to AAV and re-dosed animals produced significant anti-capsid responses against administration [118]. These findings suggest that expressing host-matched antibodies (e.g., human-derived monoclonal antibodies expressed in humans) may be advantageous over heterologous antibodies, which can be more immunogenic, and show the importance of species-matched antibody design alongside the use of low-immunogenic AAV capsids to support sustained in vivo expression.

### 4.2. Emerging Innovations for sIgA Production in Vivo

Given that sIgA is difficult to produce and deliver in vivo because of its multimeric structure, numerous new strategies have been developed to improve its expression and stability (see Table 2).

An emerging innovation in sIgA production is the introduction of mutations in heavy and light chain domains for increased stability and expression in vivo [119]. The IgA2 isotype expressed at mucosal surfaces lacks stabilizing disulfide bonds, limiting its half-life in vivo compared to the IgA1 isotype expressed in the serum [119]. Research has shown that the introduction of a proline to arginine mutation at position 221 (P221R) in the heavy chain of IgA2 allows disulfide bound formation between the heavy and light chains, resulting in higher sIgA expression yields and stabilization in Nicotiana benthamiana [119]. This work establishes a basis for employing stabilizing mutations in sIgA for in vivo production, potentially overcoming the challenges associated with its short half-life. Another recent innovation in the improvement of sIgA production involves the creation of IgA-IgG fusion antibodies for the extension of serum half-life [120]. A polymeric IgA-IgG fusion antibody was produced through the combining of the Fc regions of IgG1 and IgA2 and the incorporation of a cysteine mutation at position 242 (C242) for the deletion of the destabilizing hinge region of IgA2 [120]. The administration of this fusion antibody to mice resulted in its stabilization in the serum for up to 4 days, which is a notable improvement over recombinant IgA administration [120]. Though much work must be done to improve IgA-IgG stabilization in vivo, this research provides a promising strategy for increasing IgA half-life in vivo. Further advancements in sIgA production have focused on enhancing pIgR expression levels in animal models to improve IgA transcytosis and mucosal secretion [121]. Experiments have shown that transgenic mice overexpressing pIgR in mammary epithelial cells by up to 270-fold above basal levels produced sIgA levels up to 2-fold higher than normal levels in milk [121]. Additional findings suggested that the relationship between pIgR and sIgA expression is not proportional and a limit of natural sIgA expression is reached despite increased pIgR levels [121]. These conclusions are significant as they imply that increased pIgR levels could increase the transport and expression of vector produced sIgA beyond basal levels of natural sIgA expression. Recent studies of sIgA expression have also started to explore the use of an 46 amino acid albumin binding domain (ABD) for the extension of IgA half-life through promoting interactions with the FcRn receptor [122,123]. IgA antibodies were engineered to contain variable regions from Her2-specific IgG antibodies, and an ABD enabling these new chimeric antibodies to bind serum albumin and subsequently interact with the FcRn receptor [122]. The binding of the chimeric IgA albumin complex to the FcRn is significant as this prevents the lysosomal degradation of the antibody and allows its recycling back into the bloodstream [122]. This innovation could be crucial in extending the half-life of vector produced sIgA in vivo, which normally lacks the ability to be recycled resulting in rapid clearance. In addition, ongoing advances in sIgA production have mitigated the challenge of short half-life by engineering IgA with fewer glycosylation sites reducing receptor mediated clearance [124]. An epidermal growth factor receptor specific IgA was engineered to encode the N166 and N337 N-glycosylation site mutations, which reduced its overall glycosylation profile and limited recognition by the asialoglyprotein receptor (ASGPR) in the liver [124]. The loss of glycosylation sites in IgA prevented binding of the ASGPR and targeting for degradation resulting in increased antibody half-life in the serum of mice while preserving antigen-binding properties [124]. These findings represent an important step forward in the development of less immunogenic sIgA antibodies that retain full functionality and can be expressed in vivo for longer periods of time.

### 4.3. Considerations for Clinical Translation AAV-Vectored sIgA Expression In Vivo

**Figure 4:**
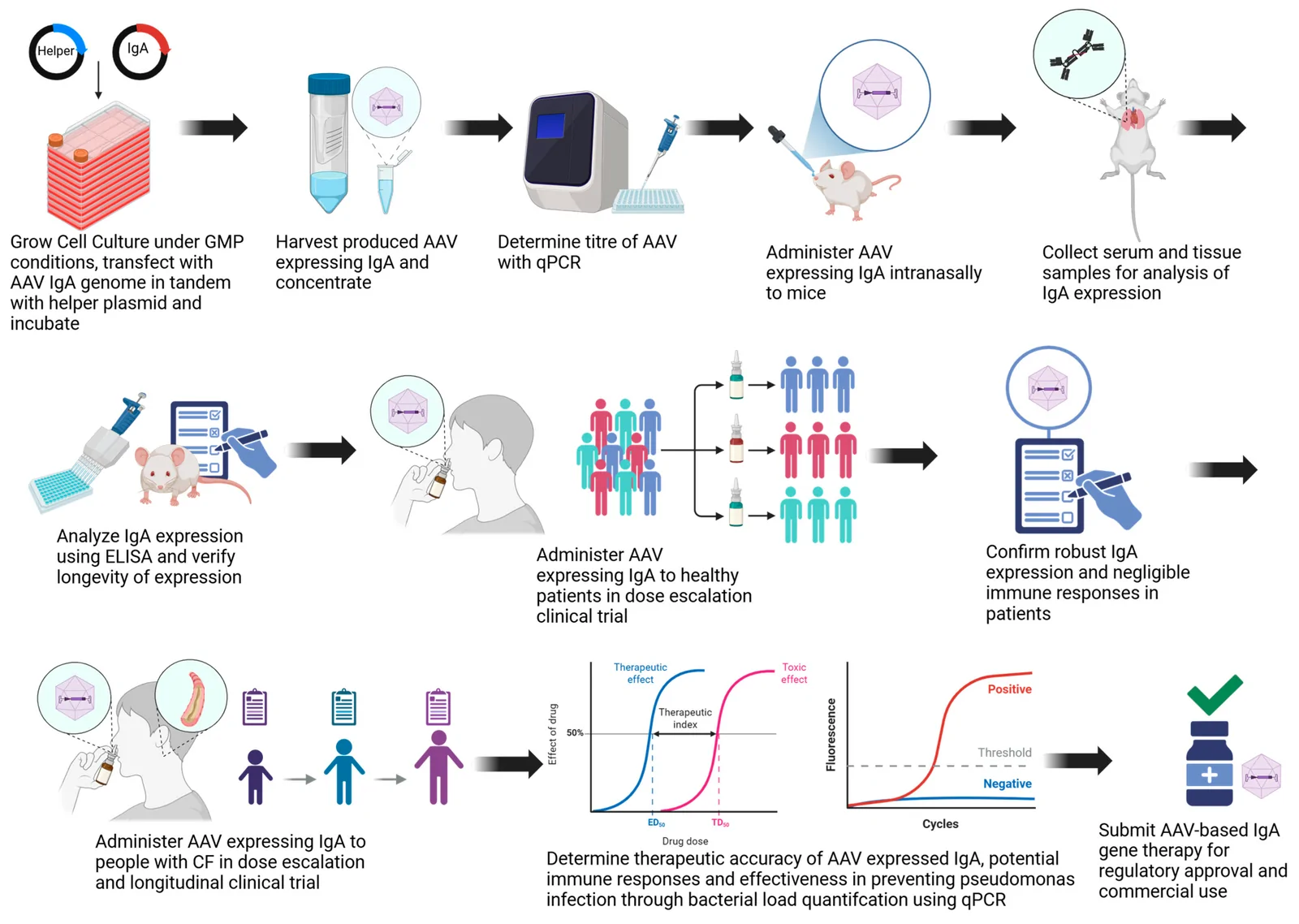
Simplified schematic of potential workflow required to produce sIgA in pre-clinical animal models, clinical translation of sIgA vectored immunoprophylaxis therapeutics for people with cystic fibrosis (CF), and regulatory approval for commercial use. Created in BioRender. Wootton, S. (2026) https://BioRender.com/8o8ys4g (accessed on 1 July 2026).

To date, there have only been two phase 1 AAV-VIP clinical trials conducted in humans with both designed to express broadly neutralizing IgG antibodies against HIV from AAV1 or AAV8 vectors. The first clinical trial involved AAV1-mediated expression of the PG9 IgG1 broadly neutralizing antibody against HIV envelope proteins [125]. This trial consisted of 21 healthy men aged 18–24 who were evaluated over 48 weeks after being IM-administered different doses of the therapeutic AAV [125]. Specifically, dose escalation was conducted in which four cohorts of participants were administered doses of the AAV ranging from 4 × 10^12^ vg to 1.2 × 10^14^ vg to determine tolerability [125]. The collection of patient serum and use in HIV neutralization assays showed that only 4 patients displayed low neutralizing activity with no PG9 IgG antibody able to be detected using ELISAs [125]. Additionally, almost all participants produced ADAs against the therapeutic antibody by week 4 and anti-capsid responses by week 6 post-AAV administration, leading to reduction of the therapeutic antibody in patient sera [125]. Interestingly, it was found that these immune responses did not readily cause harm to participants in the study and only contributed to lower efficiency of the therapeutic AAV [125]. The second clinical trial employed AAV8-mediated expression of the broadly neutralizing HIV mAb, VRC07, with dose escalation in eight participants already living with HIV [126]. Similar to the first clinical trial, it was found that serum levels of the therapeutic antibody decreased over time due to anti-capsid and ADA responses, and as before, there was no evidence of toxicity [126]. Though HIV viral loads were not reduced in participants, this clinical trial showed feasibility of AAV VIP-mediated antibody expression and tolerability in human participants [126]. Overall, these clinical trials show that VIP-mediated expression of antibodies in humans is safe and feasible and suggest that for sIgA, overcoming immune responses in patients could be a significant obstacle. For the transition of a VIP sIgA therapy to clinical trials, good manufacturing practices (GMPs) must be considered during production of AAV vectors in which preparations must be free of contaminants including cellular DNA and proteins [127]. In addition in vivo animal model studies must be conducted in the future to ensure the efficacy of an sIgA therapeutic before the transition to clinical studies and regulatory approval (see Figure 4).

### 4.4. Potential of IgA Therapeutics in Preventing Respiratory and Enteric Infections

Given the global burden of respiratory and enteric infections, IgA therapeutics offer a promising strategy to enhance mucosal immunity and prevent disease. *P. aeruginosa* is a pathogen of global importance most notably causing respiratory infections in people with cystic fibrosis (pwCF), with 98% of patients being culture positive for infection by the age of three [128]. Infection of pwCF by *P. aeruginosa* causes progressive deterioration in lung function due to the structural damage of airways leading to high mortality rates and shorter life expectancies [128]. The use of antibiotics remains one of the most effective treatments for the elimination of *P. aeruginosa* infections, though their prevalent use has caused the emergence of multi-drug resistant strains [129]. The development of live-attenuated vaccines against *P. aeruginosa* have shown success in inducing elevated levels of IgA at mucosal surfaces in clinical trials [130]. Although initial results have been promising, it is unclear if these vaccines effectively prevent *P. aeruginosa* infection and are safe for use in immunocompromised individuals such as pwCF [130]. Monoclonal antibody therapy is another potential avenue for the prevention of *P. aeruginosa* infections in pwCF with IgG antibodies shown to be effective in protecting mice against infection [131]. While recombinant mAbs are effective in preventing infection, they are often limited by having a short half-life in vivo requiring frequent re-dosing, which can cause elevated ADA responses [132]. The limitations of these previous methods have laid the groundwork for a AAV VIP approach in which IgA monoclonal antibodies can be produced directly in pwCF. The major advantage of the VIP method over antibiotic and mAb treatments is that prolonged in vivo IgA expression could be maintained in individuals without the need for repeat dosing [133]. The VIP method may represent a promising approach for use in pwCF who experience recurrent respiratory infections and would benefit from prolonged protection against pathogens such as *P. aeruginosa* [133]. As referenced earlier, the pre-clinical LNP mediated expression of sIgA in mice was shown to prevent lung colonization by *P. Aeruginosa* demonstrating the potential of this approach [83]. Moreover, clinical trials using AAV-mediated expression of IgG mAbs have shown no harmful immune responses elicited by patients [125], suggesting that recombinant sIgA-based VIP may represent a strategy for enhancing mucosal immunity in pwCF by providing localized protection at the airway surface. However, this approach has not yet been directly evaluated in pre-clinical or clinical studies in this or any patient population, and its safety, efficacy, and long-term feasibility remain to be established. Further studies will be required to determine its potential clinical utility in pwCF. Beyond bacterial respiratory infections, the VIP mediated expression of sIgA at mucosal surfaces may offer a promising approach in preventing viral infections such as influenza [134]. Studies investigating influenza infection have shown that sIgA has potent neutralizing activity at mucosal surfaces in comparison to other antibody isotypes, including enhanced blocking of viral attachment in respiratory epithelial cells and intracellular neutralization during transcytosis [134]. In addition, combining VIP-mediated expression of sIgA with existing therapies such as antibiotics may represent a future strategy to enhance protection against respiratory infections though clinical studies must be conducted to determine efficacy [129]. The *Salmonella typhimurium* serotype of *S. enterica* causes systemic infection of the GI tract resulting in the development of enteric fever which can cause serious illness in immunocompromised individuals [135]. Outbreaks of salmonella are responsible for 1.35 million illnesses each year with over 26,500 hospitalizations in the United States alone, making this pathogen of global importance [135]. Like *P. aeruginosa*, antibiotics are the most common treatment of salmonella infection, though antibiotic-resistant strains are becoming ever more prevalent [135]. Live attenuated vaccines of *S. enterica* have been proven effective in eliciting immune responses and providing protection against lethal infection in mice [136]. Nonetheless, the safety of these vaccines have not been validated in clinical trials or immunocompromised individuals most prone to infection [136]. Furthermore, the oral administration of monoclonal IgA antibodies in mice were shown to be effective in limiting invasion of Peyer’s patches in the intestines by *S. enterica* [137]. Despite these results, the use of mAb treatments in the GI tract is challenging due to the highly acidic environment, drastically limiting their half-life to only a few minutes [137]. The use of VIP for sustained IgA expression and subsequent localization to the intestines could overcome the challenges associated with oral administration by inducing mucosal surface expression and avoiding the stomach altogether. Like in *P. aeruginosa*, the pre-clinical LNP-mediated expression of sIgA at intestinal mucosal surfaces in mice was successful in preventing infection by *S. enterica* [83]. The VIP-mediated expression of sIgA could also be used for the treatment of inflammatory bowel disease (IBD) caused by imbalances in the intestinal microflora [138]. There are 2 types of IBD consisting of Crohn’s disease and ulcerative colitis with these diseases affecting approximately 0.5% of the global population [138]. As stated previously, IgA plays a critical role in the homeostasis of the intestinal microbiota by limiting the growth of potentially pathogenic microbes [139]. Studies have demonstrated that orally administered IgA antibodies targeting colitogenic bacteria in gnotobiotic mice colonized with human IBD microbiota effectively reduced gut inflammation [139]. While effective, this treatment is limited by the short half-life of mAbs in the gut meaning repeated dosing would be a requirement [139]. AAV VIP-mediated expression of IgA targeting colitogenic bacteria at intestinal mucosal surfaces could provide prolonged expression potentially limiting the need for repeated dosing. Taken together, these findings highlight the potential of VIP-mediated IgA therapeutics to provide long lasting and targeted expression at mucosal surfaces, offering a novel strategy for the prevention of mucosal infections.

## 5. Conclusions

IgA antibodies offer an advantage over IgG because they mediate immune exclusion at mucosal surfaces, preventing pathogen adherence and invasion while limiting inflammation. Their resistance to proteolytic degradation and ability to neutralize pathogens in secretions make them highly effective for mucosal immunity. The development of IgA VIP therapeutics offers a promising approach for the prevention of a broad range of respiratory and enteric mucosal infections. This novel approach has the potential to produce therapeutic IgA antibodies in vivo for extended periods of time, eliminating the need for repeated dosing as is required for recombinant mAb treatments. However, advancement of this approach has been limited by the complexity of in vivo sIgA production and the generation of host ADA and anti-AAV capsid immune responses. Hence, it is imperative that the optimal stoichiometric expression of sIgA components in vivo is achieved and host therapeutic immune responses dampened. Multiple approaches to produce recombinant IgG in vivo have been explored with great success and could be applied to sIgA, including the implementation of polycistronic expression systems, enhancer sequences, and stabilizing mutations to improve folding and secretion, as well as glycosylation site modifications to optimize function. Additionally, many different gene delivery vehicles could be employed for targeted sIgA at mucosal surfaces such as AAV, adenoviruses, lentiviruses, and LNPs. To combat therapeutic immune responses, a variety of approaches from previous studies could be applied consisting of immunosuppressants, inclusion of APC miRNA binding sites for transgene suppression, and reduction of immunoreactive epitopes of expression vectors. Addressing the production and immune related challenges of IgA VIP therapeutics will be essential for unlocking their full potential and translation to clinical studies.

## Figures and Tables

**Table 2:** Summary of recent innovations for improving sIgA production and stability.

Innovation	Engineering Approach	Mechanism of Action	Key Outcome	Reference
Stabilizing mutation in IgA heavy chain	P221R mutation introduced into IgA2 heavy chain	Enables disulfide bond formation between heavy and light chain, increasing structural stability	Increased sIgA expression yield and stability	[112]
IgA-IgG fusion antibodies	Fusion of IgG1 Fc and IgA2 Fc regions with C242 mutation to delete destabilizing IgA2 hinge	Combines IgA effector functions with IgG serum persistence and FcRn-mediated recycling	Detectable serum IgA levels for a longer period	[113]
Enhanced pIgR expression	Overexpression of pIgR in epithelial cells	Increases transcytosis of dIgA across epithelial cells	Increased sIgA expression at mucosal surfaces	[114]
Albumin-binding domain (ABD) incorporation	Incorporation of a 46 amino acid ABD to IgA antibodies	Enables albumin binding and FcRn-mediated recycling	Reduced clearance of IgA antibodies and prolonged circulation	[115,116]
Reduction of IgA glycosylation	Removal of N166 and N337 N-glycosylation sites	Prevents ASGPR-mediated clearance	Increased half-life of IgA without loss of antigen-binding efficiency	[117]

## Data Availability

No new data were created or analyzed in this study. Data sharing is not applicable to this article.

## Abbreviations

The following abbreviations are used in this manuscript:

Abbreviation	Full Name
2A	Viral 2A Self-Cleaving Peptide Sequence
AAV	Adeno-Associated Virus
AAV-mAb	Adeno-Associated Virus–Monoclonal Antibody
AAV6.2FF	Engineered Adeno-Associated Virus Serotype 6 Variant (6.2FF Capsid)
ABD	Albumin Binding Domain
ADA	Anti-Drug Antibodies
ADE	Antibody-Dependent Enhancement
AID	Activation-Induced Cytidine Deaminase
APC	Antigen-Presenting Cell
ASGPR	Asialoglycoprotein Receptor
β-actin	Beta Actin (housekeeping gene promoter element)
BiP	Binding Immunoglobulin Protein (also GRP78)
CASI	CMV Enhancer/Chicken β-Actin/Hybrid Promoter System
CD	Cluster of Differentiation
CD4^+^	Cluster of Differentiation 4 Positive (T Helper Cell Marker)
CD89	Cluster of Differentiation 89
CFU	Colony Forming Units
CHO	Chinese Hamster Ovary Cells
CMV	Cytomegalovirus (Promoter/Enhancer)
CDRs	Complementarity-Determining Regions
Cre	Cyclization Recombination Enzyme (Cre recombinase)
CSR	Class Switch Recombination
C242	Cysteine Substitution at Position 242
Dectin-1	Dendritic Cell-Associated C-Type Lectin-1
dIgA	Dimeric Immunoglobulin A
DNA	Deoxyribonucleic Acid
EGFR	Epidermal Growth Factor Receptor
ELISA	Enzyme-Linked Immunosorbent Assay
E2A	Equine Rhinitis A Virus 2A Peptide
Fab	Fragment Antigen-Binding
Fc	Fragment Crystallizable
FcαRI	Fc Alpha Receptor I
FcγR	Fc Gamma Receptor
FcGBP	Fc Gamma Binding Protein
FcRn	Neonatal Fc Receptor
F2A	Foot-and-Mouth Disease Virus 2A Peptide
GAG/POL	Group-specific Antigen / Polymerase Genes
GALT	Gut-Associated Lymphoid Tissue
GI	Gastrointestinal
GMP	Good Manufacturing Practices
GRP78	Glucose-Regulated Protein 78 *(if used separately in manuscript)*
GSG	Glycine-Serine-Glycine Linker
HGH	Human Growth Hormone
HN	Hemagglutinin-Neuraminidase
Her2	Human Epidermal Growth Factor Receptor 2
HEK293	Human Embryonic Kidney 293 Cells
HIV	Human Immunodeficiency Virus
IBD	Inflammatory Bowel Disease
IgA	Immunoglobulin A
IgA-IgG	Immunoglobulin A–Immunoglobulin G Fusion Antibody
IgA2	Immunoglobulin A2 Isotype
IgG	Immunoglobulin G
IgM	Immunoglobulin M
IM	Intramuscular
IN	Intranasal
IP	Intraperitoneal
IT	Intratracheal
ITAM	Immunoreceptor Tyrosine-Based Activation Motif
ITR	Inverted Terminal Repeats
IV	Intravenous
J-chain	Joining Chain
kb	Kilobase
kDa	Kilodalton
loxP	Locus of X-over P1 (Cre recombinase recognition site)
LNP	Lipid Nanoparticle
LPS	Lipopolysaccharide
LTB4	Leukotriene B4
LTR	Long Terminal Repeat
M2	Matrix Protein 2 (Influenza A Virus)
mAb	Monoclonal Antibody
MEDI3902	Bispecific Monoclonal Antibody
MHC II	Major Histocompatibility Complex Class II
miR-142-3p	MicroRNA 142-3p
mRNA	Messenger Ribonucleic Acid
Muc2	Mucin 2
MZB1	Marginal Zone B and B-1 Cell-Specific Protein
NADPH	Nicotinamide Adenine Dinucleotide Phosphate
NF-κB	Nuclear Factor Kappa B
NHEJ	Non-Homologous End Joining
NHPs	Non-Human Primates
N166	Asparagine 166 (amino acid position)
N337	Asparagine 337 (amino acid position)
PAO1	*Pseudomonas aeruginosa* Strain PAO1
PAMPs	Pathogen-Associated Molecular Patterns
PcrV	*Pseudomonas aeruginosa* Type III Secretion System Protein V
PD-1	Programmed Cell Death Protein 1
PD-L1	Programmed Death-Ligand 1
PDI	Protein Disulfide Isomerase
PG9	Broadly Neutralizing Antibody (HIV Envelope-Targeting Antibody)
pIgR	Polymeric Immunoglobulin Receptor
P221R	Proline-to-Arginine Substitution at Position 221
*P. aeruginosa*	*Pseudomonas aeruginosa*
Psl	*Pseudomonas aeruginosa* Polysaccharide Psl
P2A	Porcine Teschovirus-1 2A Peptide
pwCF	People with Cystic Fibrosis
rev	Regulator of Virion Expression (HIV-1 accessory protein)
RSV	Respiratory Syncytial Virus
*S. enterica*	*Salmonella enterica*
*S. typhimurium*	*Salmonella enterica* serovar *Typhimurium*
SC	Secretory Component
SIgAd	Selective Immunoglobulin A Deficiency
sIgA	Secretory Immunoglobulin A
SIN	Self-Inactivating
SIV	Simian Immunodeficiency Virus
SORT	Selective Organ Targeting
SV40	Simian Virus 40 Polyadenylation Signal
TcdA	*Clostridioides difficile* Toxin A
TcdB	*Clostridioides difficile* Toxin B
TCR	T-Cell Receptor
T2A	Thosea asigna Virus 2A Peptide
tRNA	Transfer Ribonucleic Acid
TRIM	Tripartite Motif Protein Family
TRIM21	Tripartite Motif Containing 21
vg	Vector Genomes
VIP	Vectored Immunoprophylaxis
VP6	Viral Protein 6
VRC07	Broadly Neutralizing Antibody (HIV-Targeting Monoclonal Antibody)
WPRE	Woodchuck Hepatitis Virus Post-Transcriptional Regulatory Element
